# Clinical follow-up on weight loss, glycemic control, and safety aspects of 24 months of duodenal-jejunal bypass liner implantation

**DOI:** 10.1007/s00464-019-06752-8

**Published:** 2019-03-14

**Authors:** B. Betzel, M. I. Cooiman, E. O. Aarts, I. M. C. Janssen, P. J. Wahab, M. J. M. Groenen, J. P. H. Drenth, F. J. Berends

**Affiliations:** 1 Department of Gastroenterology and Hepatology, Radboud University Medical Center, P.O. Box 9101, Code 455, 6500 HB Nijmegen, The Netherlands; 2 Vitalys Clinic, Velp, The Netherlands; 3 Department of Surgery, Rijnstate Hospital, Arnhem, The Netherlands; 4 Department of Gastroenterology and Hepatology, Rijnstate Hospital, Arnhem, The Netherlands

**Keywords:** Adverse events, Duodenal-jejunal bypass liner, Diabetes mellitus, Endobarrier, Hepatic abscess, Migration

## Abstract

**Background:**

The duodenal-jejunal bypass liner (DJBL) is an endoscopic device designed to induce weight loss and improve glycemic control. The liner is licensed for a maximum implant duration of 12 months. It might be hypothesized that extension of the dwelling time results in added value. The goals of our study were to determine weight change, change in glycemic control, and safety in patients with an intended 24 months of DJBL dwelling time.

**Methods:**

Patients were initially selected for a 12-month implantation period. When no physical complaints or adverse events (AEs) occurred, motivated patients who responded well were selected for extension of dwelling time to 24 months. Patients underwent a control endoscopy 12 months after implantation and visited the out-patient clinic every 3 months up to explantation. Patients agreed to remove the DJBL when complaints or AEs occurred that could not be treated conservatively.

**Results:**

Implantation was extended in 44 patients, and 24 (55%) patients completed the full 24 months. Twenty patients required early removal due to AEs. During dwelling time, body weight decreased significantly (15.9 kg; TBWL 14.6%). HbA1c decreased non-significantly (4.9 mmol/mol). The number of insulin users and daily dose of insulin both decreased significantly. At 24 months after removal, glycemic control had worsened, while body weight was still significantly lower compared to baseline. In total, 68% of the patients experienced at least one AE. Two patients developed a hepatic abscess.

**Conclusions:**

DJBL treatment results in significant weight loss and improves glycemic control during implantation. The largest beneficial effects occur during the first 9–12 months after implantation. Extension of dwelling time to 24 months results only in stabilization of body weight and glycemic control. After explantation, weight improvements are maintained, but glycemic control worsens. As the cumulative risk of AEs increases with time, a maximal dwelling time of 12 months is advisable.

**Electronic supplementary material:**

The online version of this article (10.1007/s00464-019-06752-8) contains supplementary material, which is available to authorized users.

The increasing prevalence of obesity calls for the development of weight control measures. The current mainstay of treatment for morbid obesity is bariatric surgery. Roux-en-Y gastric bypass is one of the most performed bariatric procedures and has proven to be effective in inducing weight loss and controlling comorbidities such as type 2 diabetes mellitus (T2DM) and hypertension [1, 2]. As surgery carries the risk of intra- and postoperative adverse events (AEs), there is great interest for less definitive solutions. The duodenal-jejunal bypass liner (DJBL) is an endoscopic device that has been developed to mimic the effect of a gastric bypass [3]. The endoscopically placed DJBL prevents direct contact of nutrients with the duodenum and proximal jejunum. This results in metabolic changes that induce weight loss and improve glycemic control. Since not all patients are eligible for bariatric surgery or willing to undergo surgery, the DJBL might be a therapeutic option for these patients. The advantage of the DJBL is that it can be removed at any time point without leaving any change in original anatomy. Early studies employed an implantation period of 3 months and gradually stretched dwelling time to 12 months. It was hypothesized that extension of the implantation period would result in additional weight loss and further improvement of diabetic and cardiovascular parameters. Furthermore, it might be possible that an increased implantation period results in long lasting positive effects that extend beyond removal. Safety and efficacy data that report on longer implantation periods could assist in clinical decision making since longer implantation of a medical device in the small intestine potentially holds the risk of an increased AE rate. Because of its current safety profile, the DJBL is not approved by the Food and Drug Administration (FDA) and is therefore not allowed for use in the United States. As the primary implantation site in Europe, we assembled a cohort to examine the effect of long term (> 12 months) implantation. The primary goals of our study were to determine efficacy, measured as weight change and change in glycemic control, as well as safety in patients with an intended 24 months of DJBL implantation. As secondary endpoint, we studied changes in weight and glycemic parameters after removal of the DJBL.

## Methods

### Patient selection

**Fig. 1:**
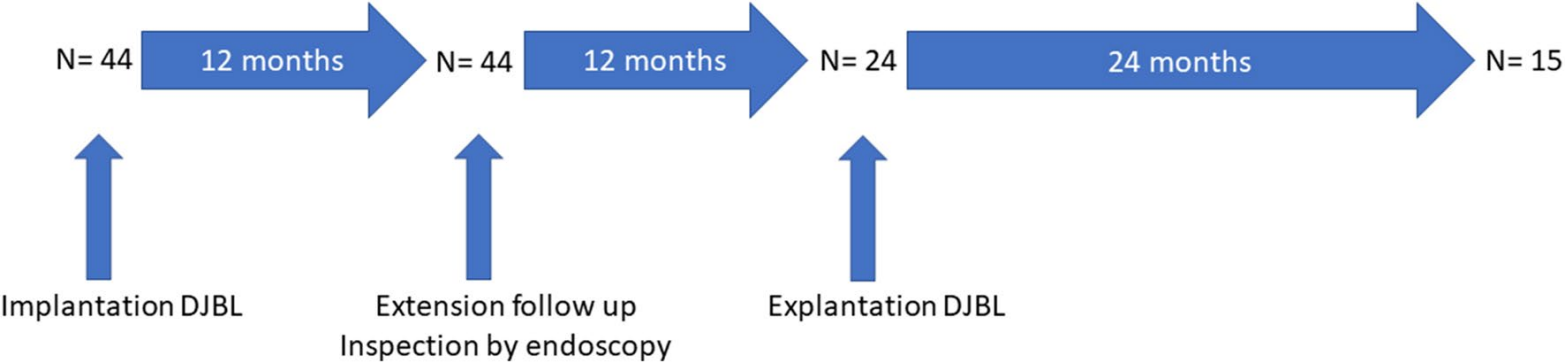
Flow chart of study protocol

All patients were initially selected for a 12-month implantation period with DJBL [4]. The major inclusion criteria consisted of age 18–70 years, BMI 28–45 kg/m^2^, and type 2 diabetes mellitus (T2DM) with glycosylated hemoglobin A_1c_ (HbA1c) levels > 48 mmol/mol. The major exclusion criteria were use of non-steroidal anti-inflammatory drugs or anticoagulant medication. After 9 months of implantation, patients were selected for this prospective cohort study with an extended implantation time to 24 months when they met and agreed to the following criteria (Fig. 1): no physical complaints or AEs that are, or might be, related to the DJBL present during the first 12 months of implantation; consent to undergo an endoscopy 12 months after implantation to evaluate the anatomical position of the DJBL and in case abnormalities are present, consent to removal of DJBL; out-patient clinic visits every 3 months with laboratory tests up to explantation; agree to report physical complaints immediately to the treating physician and allow early removal of the DJBL when the complaints cannot be treated conservatively; and received informed consent on the potential increased risks of prolonged implantation such as migration, ulceration, and hemorrhage. Finally, motivated patients were selected who showed a decrease in weight and diabetes parameters during the first 9 months of treatment. After explantation, follow-up continued for at least 12 additional months.

The extension of the implantation period was approved by the local institutional review board and the research ethics committee of Nijmegen, The Netherlands (Registration Number 2013/510).

### Definitions

We analyzed changes in weight as change in absolute weight, BMI, and total body weight loss (TBWL). HbA1c is expressed as mmol/mol. The following formula can be used to change the unit mmol/mol to percentage: (0.0915 × HbA1c in mmol/mol) + 2.15%. We used analysis of change in HbA1c and changes in anti-diabetic drugs to determine T2DM regulation. Changes in anti-diabetic drugs were analyzed as percentage of use over time and changes in dosages were compared with initial use.

AEs were classified as mild, moderate, or severe according to the severity grading system defined by the American Society of Gastrointestinal Endoscopy (ASGE) [5].

### Statistical analysis

IBM SPSS Statistics for Windows, version 23.0 (Armonk, NY, IBM Corp.) was used for statistical analyses. Continuous variables are reported as mean ± standard deviation (SD) unless specified otherwise. Since Bonferroni correction was used for multiple testing, a *p* value of < 0.017 was considered statistically significant. Since data were not normally distributed, analyses between different time points were conducted with a Wilcoxon signed-rank test or McNemar’s test.

To deal with missing data because of early explantations, data were analyzed using last observation carried forward until the time point of 24 months after implantation was reached.

Figures were created using GraphPad Prism version 5.03 (GraphPad Software, Inc.).

## Results

### Patient selection and baseline characteristics

In total, 44 patients had their implantation period extended with an intended period of 24 months between June 2013 and January 2018. The mean dwelling time was 22.0 ± 4.3 months. At time of screening, the 44 included patients had a mean age of 58 (± 7.2) years of which 52% was female (Table 1). At time of screening, mean body weight was 108.3 kg (± 17.3), compatible with a BMI of 35.1 kg/m^2^ (± 4.4). All patients suffered from T2DM with a mean duration of 10 years and a mean HbA1c of 67 mmol/mol (± 16.5). The majority of the patients were treated at time of screening with metformin (84%) and 59% used insulin with a mean dose of 95 IU (± 62) each day.

**Table 1:** Baseline characteristics at time of screening for patients with extension of the intended dwelling time to 24 months

	*N* = 44
Age (years)	58 ± 7.2
Female	23 (52.3%)
Body weight (kg)	108.3 ± 17.3
BMI (kg/m^2^)	35.1 ± 4.4
Duration T2DM (years)	10.1 ± 6.4
Blood values	
HbA1c (mmol/mol)	67 ± 16.5
Fasting glucose (mmol/L)	10.7 ± 3.5
Anti-diabetic drugs (% users and dosage)	
Metformin (mg)	84% (1975 ± 690)
Glimepiride (mg)	34% (4.7 ± 2.2)
GLP-1 agonist (mg)	11% (2.2 ± 0.8)
Insulin (IU)	59% (95 ± 62)

Twenty-four (55%) patients completed the full implantation period and 20 (45%) patients required premature explantation.

### Body weight

**Fig. 2:**
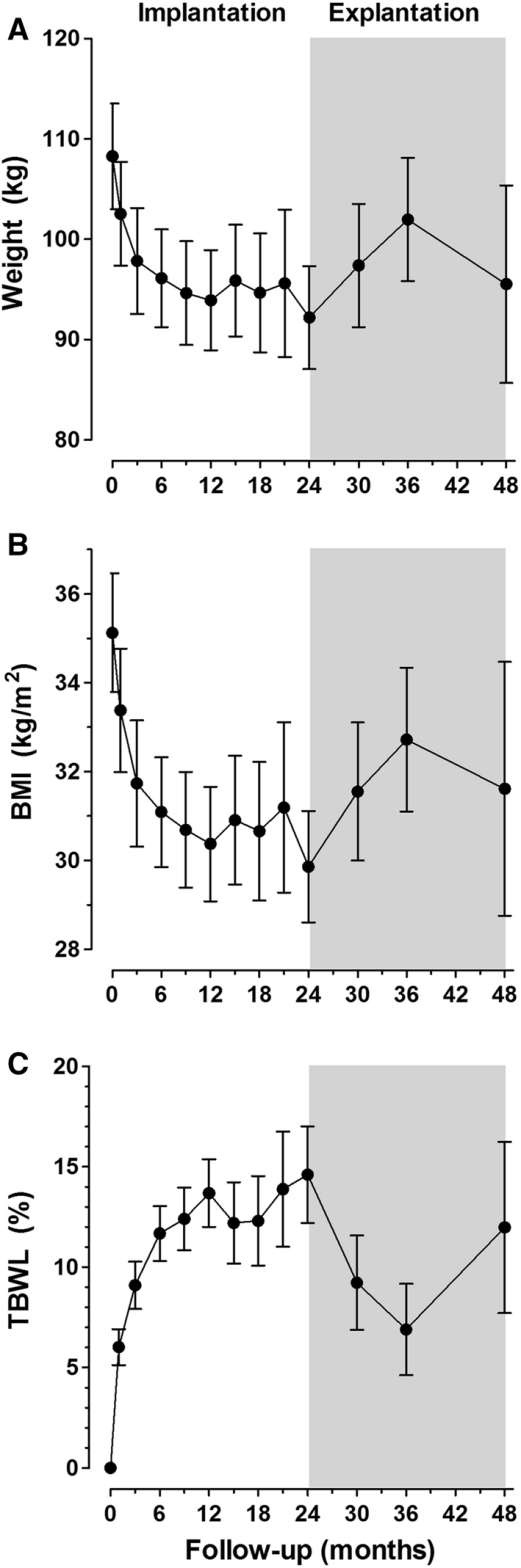
Mean changes with 95% confidence interval in weight, BMI, and TBWL during DJBL dwelling time and after explantation

Figure 2 shows mean changes in weight, BMI, and TBWL during DJBL dwelling time and after explantation. There was a mean weight loss of 15.9 kg (± 9.3) (*p* < 0.001) between start of implantation and explantation, that is comparable to a TBWL of 14.6% (± 7.8) (*p* < 0.001). Twelve months after explantation there was weight regain of 6.5 kg (± 5.6) or a TBWL increase of 6.9% (± 5.3) (*p* < 0.001). However, 24 months after explantation weight had decreased again with 13.2 kg (± 9.0) or 12.0% (± 7.7) TBWL, and was still significantly lower compared to baseline (*n* = 15).

### Type 2 diabetes mellitus

**Fig. 3:**
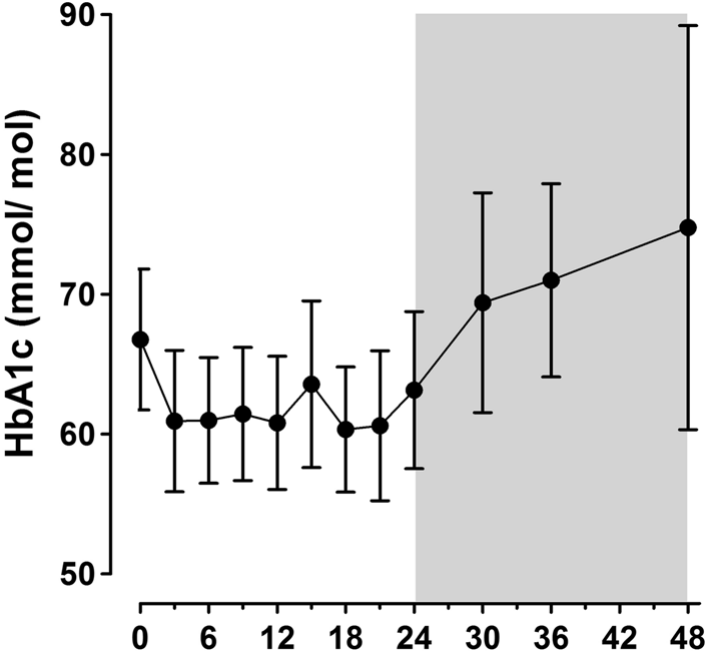
Mean change with 95% confidence interval of HbA1c during DJBL dwelling time and after explantation

After implantation of the DJBL, HbA1c decreased sharply during the first 3 months and bottomed off during the dwelling time, with a mean decrease after 24 months of 4.9 mmol/mol (± 16.7) (*p* = 0.087). Compared with explantation, HbA1c rose with 6.9 mmol/mol (± 18.5) (*p* = 0.085) 12 months after explantation, and with 15.3 mmol/mol (± 29.1) to 74.8 mmol/mol (± 23.9) (*p* = 0.059) 24 months after explantation (Fig. 3).

We observed several changes in composition and dose of anti-diabetic drugs (Supplementary File 1). The majority of medication number and dosage analysis 24 months after explantation were not possible because of the low numbers per group due to lost to follow-up and scheduled visits in the future. During the DJBL dwelling time, 11 patients could stop their insulin while its use in the remaining 14 patients decreased with 62 IE ± 72 per day (*p* = 0.007). However, at 12 months after explantation, the daily dosage of insulin had increased again by 20 IE ± 25 (*p* = 0.028) per day compared to explantation. By contrast, the number of glimepiride users and its dosage rose, albeit not significantly, during DJBL dwelling time; 15 versus 20 users, and an increase of 3.5 mg ± 1.7 per day (*p* = 0.319). The number of metformin users and their dosage remained similar during DJBL dwelling time.

One of the 44 patients reached complete resolution of T2DM. This was reached as of 3 months after implantation till explantation (37 months after implantation). At the time of screening, patient had a starting HbA1c of 49 mmol/mol, a BMI of 32.8 kg/m^2^, and used two types of oral anti-diabetic drugs. At the time of explantation her BMI had decreased to 27.2 kg/m^2^ (TBWL 17.1%), her HbA1c to 41 mmol/mol, and she had stopped all of her anti-diabetic drugs.

### Safety

**Fig. 4:**
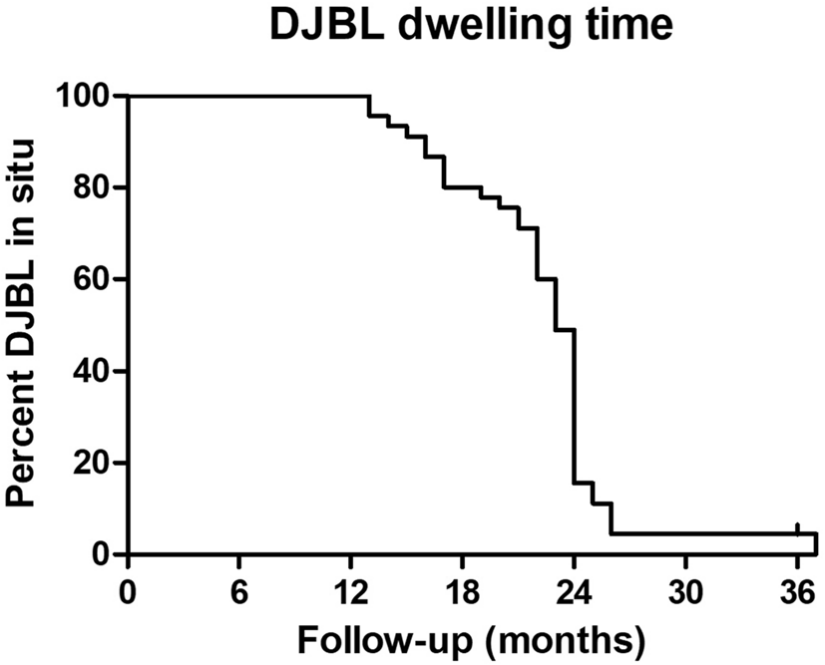
Follow-up of DJBL dwelling time

In total, 20 (45%) patients required early removal due to AEs, starting as early as 1 month after extension of the dwelling time (Fig. 4). Thirty (68%) patients reported at least one AE. These 30 patients reported 49 AEs in total, ranging from mild to severe (Table 2). No patients died. Two patients (4.1%) developed a hepatic abscess 14 and 17 months after implantation and were classified as severe AEs. In three (6.5%) patients, mechanical failure of the device was observed, in which the sleeve of the DJBL was disconnected from the anchor. In one patient, the DJBL was removed 37 months after implantation since she refused to explant the DJBL in spite of signed informed consent. Ultimately, the DJBL was removed because of abdominal complaints.

**Table 2:** Adverse events during dwelling time and after explantation

Mild AEs	
Mucosal laceration esophagus^a^	1
Reflux esophagitis Los Angeles grade A	1
Sleeve disconnected from anchor	3
Moderate AEs	
Mucosal laceration esophagus	1
Gastrointestinal events (e.g., abdominal pain, nausea, vomiting)	13
GI hemorrhage	1
GI hemorrhage after explantation	1
Anemia^b^	1
Migration (> 5 cm) of DJBL	8
Partial migration (< 5 cm) of DJBL	11
DJBL anchor tissue overgrowth	1
DJBL anchor perforation^c^	1
Dilatation pylorus required to pass pylorus	1
Obstruction liner with food	1
Two explantation procedures required^d^	2
Severe AEs	
Hepatic abscess	2
Total	49

^a^No admission required

^b^Four days prior to anemia patient underwent abdominal wall reconstruction with active bleeding out of two drains

^c^Presentation 36 months after DJBL implantation

^d^Unable to pass the esophagus due to a stenosis in the esophagus and inability to pass the lower esophageal sphincter

### Bariatric surgery

Prior to implantation, 17 (39%) patients met the eligibility criteria for BMI and diabetes to undergo bariatric surgery. The remaining 27 patients had a BMI < 35 kg/m^2^. At the time of explantation only seven patients (16%) met the eligibility criteria. Twelve months after explantation, the number of patients eligible for bariatric surgery had increased to 11 patients (37% - no data available of 14 patients).

In total, four patients underwent Roux-en-Y gastric bypass surgery after explantation of the DJBL and one additional patient was still waiting for surgery.

## Discussion

DJBL implantation results in weight loss and improvement of glycemic control as of implantation. The reached decrease in body weight and glycemic control after 12 months of implantation could be maintained during the increased dwelling time. The largest improvements are observed during the first 9–12 months after implantation. At the time of explantation, average weight loss per patients was almost 16 kg (TBWL 14.6%). In this period, HbA1c showed a non-significant decreasing trend of 4.9 mmol/mol. The number of patients requiring insulin and the daily dose of patients still on insulin both decreased significantly during dwelling time. During the extension of the dwelling time, over two-thirds of the patients experienced at least one AE, and 45% of the patients required early removal of the DJBL due to AEs. Twelve months after removal of the DJBL, clinical relapse occurred with weight regain (6.5 kg), which had improved again 48 months after explantation. A sharp increase of HbA1c and daily dose of insulin was seen compared with time of explantation.

Our data demonstrate that the biological effects of the DJBL are reached within 9 to 12 months after implantation. The desired biological effects stabilize after the first 12 months after implantation and DJBL removal results in weight regain and loss of glycemic control. This accords with data from a study with DJBL dwelling times up to 3 years [6]. A possible reason for the observed stabilization during dwelling time might be that the DJBL is tolerated better over time, patients have less abdominal complaints, early satiety diminishes, and therefore intake is increased. The weight change after removal, which shows an initial substantial increase in the first 12 months after explantation, followed by improvements in the second 12 months, is more difficult to understand. This might be explained by a new weight plateau that has been reached by the DJBL. However, this could also be explained by potentially confounding factors, such as selection bias. The results on glycemic control depend on the presence of the DJBL in the gastrointestinal tract, as removal results in complete reversal of the beneficial effects. It can be hypothesized that initial changes in incretins after implantation revert to levels seen prior to implantation when the DJBL is removed. Additionally, the possible achieved restricted food intake disappears after removal. The substantial relapse of all parameters that occurs is also seen in other studies [7, 8]. This indicates, at least for glycemic control, that only temporary suppression during implantation is possible as opposed to cure of disease.

Although relapse occurs after explantation, weight is still improved 48 months after explantation and patients have experienced a temporarily improvement in glycemic control. Without implantation of the DJBL, the expected natural course would be further weight gain and deterioration of their T2DM. Therefore, when our study population would have been compared with a control group without any intervention, the changes in weight and glycemic control might have been larger when just comparing the results within patients between explantation and baseline. A possibility to preserve achieved improvement in body weight and glycemic control and to diminish risks of long-term implantation, is explantation of the DJBL after 12 months, followed by reimplantation of the DJBL after a short time of removal. However, feasibility was tested only in a small case series [9]. Additionally, costs will increase due to the need of an extra device and implantation and explantation procedure.

Patients were selected for 24 months implantation when they tolerated the DJBL well during the first 12 months. In this cohort, 31% of the patients required early explantation during the first year of implantation, mainly caused by intolerability and AEs [4]. Moreover, the risk of AEs continuously increased during the second year. In this selection of well responders, still 45% of the patients required early removal after extension of the implantation period due to persisting abdominal complaints or AEs. In total, over two-thirds of the patients experienced an AE. Additionally, two patients developed a SAE and required prolonged hospitalization and intensive treatment as a result of a deep hepatic abscess. The cohort study of Quezada et al. describes an early removal rate of 86% before 36 months. In total, 29% of the early removals were due to AEs and 55 DJBL-related SAEs were observed in 80 patients of which three were hepatic abscess [6]. This suggests that all patients are at risk to develop (S)AEs during the complete dwelling time. Moreover, the occurrence of such hepatic abscess in patients with a DJBL resulted in early termination of a large clinical trial in the United States.

An alternative endoscopic bariatric therapy is the intragastric balloon (IGB). When combined with conventional treatment, IGB results in a mean weight loss of 14.7 kg (TBWL 12.2%) after 6 months of therapy in a large meta-analysis. Early balloon removal occurred in 4.2% of the patients [10]. Another meta-analysis saw a mean decrease of HbA1c of 9% compared to baseline and a SAE rate of 1.3% that included five cases of gastric perforation with two patients who died (mortality rate 0.04%) [11]. This same meta-analysis showed a mean weight loss of 13.5 kg (*p* < 0.001) 6 months after IGB removal, comparable with 4.8 kg/m^2^ BMI loss. A retrospective analysis of 114 patients implanted with an IGB for 6 to 12 months, found a BMI reduction of 4.1 kg/m^2^ 1 year after IGB removal [12]. Both devices, DJBL and IGB, have a similar efficacy profile during and after use. Early removal and the SAE rate of the IGB are certainly lower compared to the DJBL, although recent literature shows multiple case reports of SAEs due to IGB use, such as gastric perforation and severe bleeding [13, 14]. In contrast to the IGB, that has been implicated in 33 deaths, no mortality has been reported so far with DJBL [15, 16].

This study has several strengths and limitations. With this study, we present one of the first clinical real-world data of DJBL implantation over 12 months. Additionally, we show clinical outcomes after explantation of the DJBL. Moreover, we provided safety data of long-term implantations that are much needed for clinical decision making. This study also had several limitations. First of all this study is an observation cohort study. As with any observational study, a placebo control group was not included; therefore, this study cannot account for the natural course of obesity and diabetes over time. Secondly, responder rates should be interpreted with caution because patients who did not respond to or did not tolerate DJBL withdrew from the trial, resulting in inflated response rates. Additionally, since motivated patients were selected during the first 9 months of treatment, the presented results might be an overestimation of the true effect in a non-selected population. Finally, there was a large number of lost to follow-up 24 months after explantation, leading to bias and an overestimation of the results.

In conclusion, implantation of the DJBL results in significant weight loss and improved glycemic control. The largest beneficial effects are seen during the first 9 to 12 months after implantation. Further extension of implantation results only in stabilization of the parameters as long as the DJBL is in situ. However, after explantation relapse is seen in glycemic control. Although body weight shows also initial relapse after explantation, it is still significant improved 48 months after removal. The cumulative risk of developing AEs increases with extended dwelling time with potentially high severity. Therefore, we advise on basis of our results not to extent the dwelling time over 12 months until a better efficacy–safety margin is achieved.

## Electronic supplementary material

Below is the link to the electronic supplementary material.

Supplementary material 1 (DOCX 16 KB)

## Abbreviations

AE: Adverse event
ASGE: American Society of Gastrointestinal Endoscopy
DJBL: Duodenal-jejunal bypass liner
IGB: Intragastric balloon
SD: Standard deviation
T2DM: Type 2 diabetes mellitus
