# Supplementary material for: Clinical follow-up on weight loss, glycemic control, and safety aspects of 24 months of duodenal-jejunal bypass liner implantation
Source: Surg Endosc. 2019 Mar 14;34(1):209–15. doi: 10.1007/s00464-019-06752-8 (PMC6946747; doi:10.1007/s00464-019-06752-8)
Supplement: Supplementary file 1 — Supplementary material 1 (DOCX 16 KB) [file 464_2019_6752_MOESM1_ESM.docx]

**Supplementary file 1.** Characteristics and mean changes at explantation and 12 and 24 months after explantation

|  | **Explantation*** | **12 months post explantation*** | **24 months post explantation*†** |
| --- | --- | --- | --- |
| Weight (kg)  ∆ Screening  ∆ Explantation | 92.2 ± 16.6 (n = 43)  - 15.9 ± 9.3; p < 0.001 | 102.0 ± 16.4 (n = 30)  + 6.5 ± 5.6; p < 0.001 | 95.5 ± 17.8 (n = 15)  - 13.2 ± 9.0; p < 0.001  + 6.6 ± 5.5; p < 0.001 |
| ∆ BMI (kg/ m^2^)  ∆ Screening  ∆ Explantation | 29.9 ± 4.1 (n = 43)  - 5.2 ± 3.0; p < 0.001 | 32.7 ± 4.3 (n = 30)  + 2.1 ± 1.9; p < 0.001 | 31.6 ± 5.2 (n = 15)  - 4.3 ± 3.0; p < 0.001  + 2.3 ± 3.0; p < 0.001 |
| ∆ TBWL (%)  ∆ Screening  ∆ Explantation | 14.6 ± 7.8 (n = 43)  + 14.6 ± 7.8; p < 0.001 | 6.9 ± 6.1 (n = 30)  - 6.0 ± 5.3; p < 0.001 | 12.0 ± 7.7 (n = n = 15)  12.0 ± 7.7; p < 0.001  - 6.1 ± 5.0; p < 0.001 |
| ∆ HbA1c (mmol/ mol)  ∆ Screening  ∆ Explantation | 63.2 ± 17.3 (n = 39)  - 4.9 ± 16.7; p = 0.087 | 71.0 ± 18.2 (n = 25)  + 6.9 ± 18.5; p = 0.085 | 74.8 ± 23.9 (n = 13)  + 7.9 ± 19.2; p = 0.142  + 15.3 ± 29.1; p = 0.059 |
|  |  |  |  |
| **Anti-diabetic drugs** | | | |
| Metformin |  |  |  |
| No. of users  ∆ Screening  ∆ Explantation | 37  0; p = 1.000 | 26  -11; p = 0.250 | 14  - 23; p = 0.625  - 23; p = 0.625 |
| Dosage (mg)  ∆ Screening  ∆ Explantation | 1940 ± 870  - 65 ± 810; p = 0.714; n = 36 | 2230 ± 600  + 100 ± 510; p = 0.270; n = 26 | 2340 ± 590  + 250 ± 600; Analysis not possible  + 300 ± 510; Analysis not possible |
| Glimepiride |  |  |  |
| No. of users  ∆ Screening  ∆ Explantation | 20  + 5; p = 0.441 | 13  -7; p = 0.625 | 4  - 11; p = 1.000  - 16; p = 0.125 |
| Dosage (mg)  ∆ Screening  ∆ Explantation | 3.5 ± 1.7  - 1.5 ± 2.5; p = 0.319; n = 4 | 3.7 ± 1.9  + 0.3 ± 0.8; p = 0.166; n = 12 | 6.5 ± 1.9  N/A; Analysis not possible  + 4.5 ± 2.1; Analysis not possible |
| GLP-1 agonist |  |  |  |
| No. of users  ∆ Screening  ∆ Explantation | 6  + 1; p = 1.000 | 4  + 2; p = 1.000 | 4  - 1; p = 0.688  - 2; p = 1.000 |
| Dosage (mg)  ∆ Screening  ∆ Explantation | 2.2 ± 0.9  0.0 ± 0.0; Analysis not possible | 3.0 ± 0.0  0.0 ± 0.0; Analysis not possible | N/A; Analysis not possible  0.0 ± 0.0; Analysis not possible |
| Insulin |  |  |  |
| No. of users  ∆ Screening  ∆ Explantation | 15  -11; p = 0.001 | 11  -4; p = 0.125 | 3  - 23; p = 0.000  - 12; p = 0.000 |
| Dosage (IU)  ∆ Screening  ∆ Explantation | 53 ± 31  - 62 ± 72; p = 0.007; n = 14 | 77 ± 41  + 20 ± 25; p = 0.028; n = 10 | 92 ± 49  - 50 ± 76; Analysis not possible  + 66 ± 37; Analysis not possible |

*** Statistical analyses were performed with the Wilcoxon Signed Rank test and the McNemar test. A *p* value < 0.017 was considered statistically significant

† Follow-up complete (n = 15); lost to follow-up (n = 13); underwent Roux-en-Y gastric bypass (n = 4); follow-up visit scheduled in future (n = 12)
